# Total thyroidectomy in HIV positive patient with buffalo hump and taurine neck

**DOI:** 10.1016/j.ijscr.2019.07.020

**Published:** 2019-07-19

**Authors:** Elena Carrese, Uliano Morandi, Alessandro Stefani, Beatrice Aramini

**Affiliations:** Division of Thoracic Surgery, Department of Medical and Surgical Sciences for Children and Adults, University Hospital of Modena, Via Largo del Pozzo n. 71- 41124 Modena, Italy

**Keywords:** HIV, human immunodeficiency virus, HAART, highly active anti-retroviral therapy, AZT, zidovudine, Ddi, didanosine, d4T, stavudine, 3TC, lamivudine, IDV, indinavir, EFV, efavirenz, BMI, body mass index, HCV, hepatitis c virus, ASA, American society of anesthesiologists, PIs, protease inhibitors, NRTIs, nucleoside reverse transcriptase inhibitors, LDL, low-density lipoprotein, HDL, high-density lipoprotein, non-NRTIs, non-nucleoside reverse transcriptase inhibitors, Thyroidectomy, Lipodystrophy, Highly active anti-retroviral therapy (HAART), HIV positive

## Abstract

**Background:**

The authors present a case of multinodular goiter in an HIV-positive patient affected by lipodystrophy with particular accumulation of adipose tissue in the region of the neck and trunk.

**Case presentation:**

The patient, a 53-year-old man, presented with multinodular struma with partial retrosternal engagement, as well as multiple thyroid nodules increasing in size; some of the nodules had suspicious characteristics on ultrasound. Needle aspiration biopsy was difficult to use to determine the presence of lipodystrophy; however, even in the absence of cytology, surgical treatment was necessary due to the presence of dyspnea during exercise, the dimension of the goiter with retrosternal engagement, and the ovalization of the tracheal lumen. The patient underwent total thyroidectomy by anterior cervicotomy with particular attention to patient positioning because of the buffalo hump and taurine neck. Histological examination was positive for adenomatous hyperplasia with outbreaks of papillary microcarcinoma.

**Conclusions:**

The aim of this case report was to highlight the importance of the perioperative teamwork, with particular attention to patient positioning before surgery, as well as professional collaboration and experience among the operators.

## Highlights

• A multinodular goiter in an HIV-positive with lipodystrophy, buffalo hump and taurine neck.
• Needle aspiration biopsy was difficult to use to determine the presence of lipodystrophy.
• The goiter was with retrosternal engagement and the ovalization of the tracheal lumen.
• Surgical treatment was necessary due to the presence of dyspnea during exercise.
• Importance of the perioperative teamwork, in particular to the patient positioning.

## 1 Background

Highly active anti-retroviral therapy (HAART) with associated lipodystrophy syndrome refers to a serious metabolic syndrome in HIV-infected patients receiving highly antiretroviral therapy [4]. It is generally characterized by metabolic changes such as the development of dyslipidemia, insulin resistance, glucose intolerance and abnormal redistribution of body fat (peripheral lipoatrophy and lipohypertrophy) [3]. Lipohypertrophy refers to localized abnormal fat accumulation and most commonly occurs in the intra-abdominal compartment (visceral adipose tissue), breast, dorso-cervical area (buffalo hump) or discretely accumulates under the skin (lipomas). Lipoatrophy is characterized by loss of subcutaneous fat, particularly in the face, buttocks, arm and leg. The coexistence of lipohypertrophy and lipoatrophy is referred to as mixed lipodystrophy [5]. This syndrome was identified in 1998, but its causes are not fully understood; current data suggest a multifactorial pathogenesis, with the major contributing factors being the choice of treatment, duration of treatment and patient-related factors, such as age, BMI, and the level of immunodeficiency [3,5,4].

The aim of this report was to show perioperative management in an HIV patient affected by lipodystrophy syndrome who underwent thyroidectomy for a multinodular goiter. The work has been reported in line with SCARE criteria has been reported in line with the SCARE criteria [6].

## 2 Case presentation

**Fig. 1:**
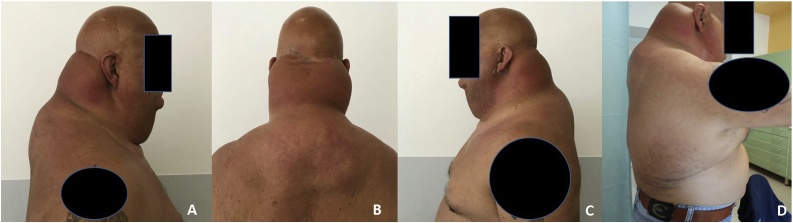
A-C-D show a lateral view of the patient affected by lipodystrophy with particular accumulation of adipose tissue in the region of the neck and trunk. Fig. 2B shows a posterior view.

We present a case of a 53-year-old man who came to our department for a large multinodular goiter with multiple suspicious nodules on ultrasound. The diagnosis was made in 1999 during hospitalization for an episode of thyrotoxicosis. The patient was diagnosed as HIV-positive in 1994 and began antiretroviral treatment with AZT (zidovudine) and Ddi (didanosine) with minimal initial benefit; however, subsequent worsening of the immunological condition was noted. Therefore, beginning in June 1997, therapy was switched to d4T (stavudine) plus 3TC (lamivudine) and IDV (indinavir); this achieved excellent viral replication control and improved the immunological condition. In 1999, a change in HIV treatment was necessary due to the presence of an important lipid dysmetabolism with hypertriglyceridemia, modification of the habitus with an important lipohypertrophy of the cervicodorsal region (buffalo hump and taurine neck) and loss of subcutaneous fat of the legs (Fig. 1A–D). Therapy with AZT (zidovudine)+ 3TC (lamivudine) + EFV (efavirenz) enabled control of viral replication and stability of the lipodystrophic picture. The patient had undergone multiple liposuctions, but they were not effective, likely due to the excessive accumulation of adipose tissue. In our opinion, the most relevant clinical aspect to consider was the large buffalo hump and taurine neck due to lipodystrophy. He also presented with negative thyroid autoimmunity, with normal calcitonin and calcemic blood values. The ultrasound exam showed struma with partial retrosternal engagement and multiple thyroid nodules increasing in size relative to a previous check, as well as suspicious features for neoplasia. The patient had undergone several needle aspirations, which failed due to the thickness and abundancy of the adipose tissue.

**Fig. 2:**
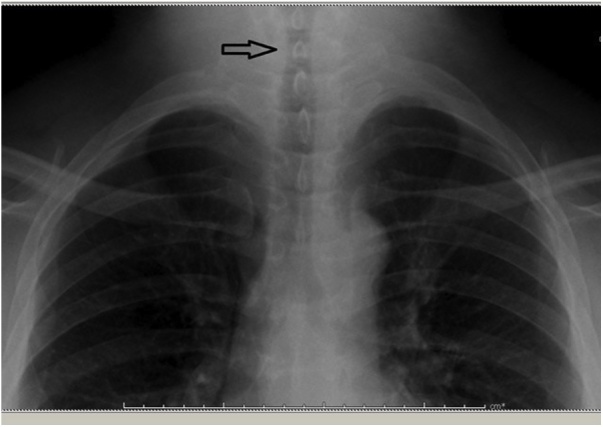
Chest x-ray shows the ovalization of the tracheal lumen (arrow).

Patient comorbidities were mainly based on the alteration of glucose metabolism (due to diabetes mellitus), alteration of bone metabolism (due to osteopenia), severe obesity (body mass index, BMI, of 34.6), and hypertension, as well as slight limitation in running, weightlifting and playing intense sports. He also reported that a previous HCV hepatopathy had been treated with Harvoni. The indication to remove the thyroid glands was suggested by the patient’s endocrinologist, primarily due to the suspicious nodules. Preoperative anesthesia evaluation predicted an American Society of Anesthesiologists (ASA) anesthetic risk of 3, requiring complex intubation for the patient’s habitus and for the shrinkage of the trachea. Indeed, preoperative chest X-ray showed an ovalization of the tracheal lumen (11 mm) to a plane passing through C7 (Fig. 2).

**Fig. 3:**
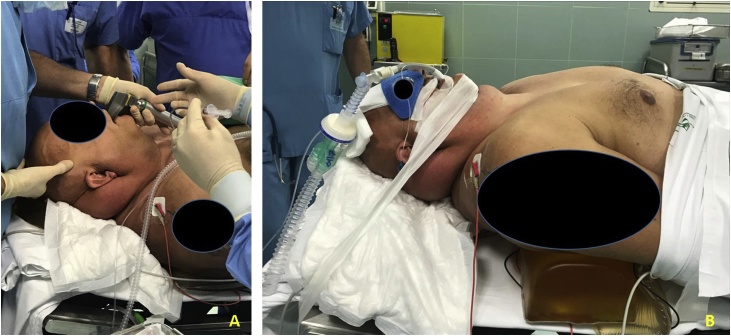
shows the patient positioning before surgery and the anesthesiologist management during intubation.

After careful positioning (Fig. 3A and B), the patient underwent total thyroidectomy by anterior cervicotomy. Total thyroidectomy was performed with technical difficulties: first, intubation of the patient was required for the operation due to the hyperextension of the neck; second, access to the goiter was difficult due to the amount of adipose tissue, as well as the buffalo hump and taurine neck. Thyroid isolation was complicated on the right side of the gland for the increased volume, particularly due to difficulty in recurring laryngeal nerve isolation. No intraoperative complications and no bleeding loss were observed during or after surgery. The drains were removed on the second postoperative day. Despite the patient’s HIV infection, no complications were noted after surgery, especially around the wound. Calcium blood values stayed in the normal range after surgery. The patient presented with dysphonia; therefore, direct laryngoscopy was performed, which showed fixation of the right vocal string with good phonatory compensation, as well as consistency of the respiratory space. Therefore, the patient was sent to logopedic rehabilitation after discharge from the hospital.

## 3 Discussion and conclusion

Lipodystrophy syndrome is an important complication of antiretroviral therapy due to its increased cardiovascular risk as a result of metabolic alterations, as well as the psychological aspects and quality of life.

Studies have shown that protease inhibitors (PIs) and some nucleoside reverse transcriptase inhibitors (NRTIs), especially stavudine (d4T), increase the levels of triglycerides, total cholesterol and low-density lipoprotein cholesterol (LDL); however, they reduce high-density lipoprotein (HDL) levels. In contrast, regimens with nevirapine and efavirenz (non-NRTIs, or NNRTIs) show reduced atherogenic effects on lipid profiles [3,1]. PIs and NRTIs appear to cause mitochondrial toxicity, modifying the enzyme activity involved in metabolism [7,8]. Other studies support a direct role for HIV: adipose tissue may serve as reservoir for HIV, altering the local tissue environment and promoting enhanced adipose tissue inflammation. HIV proteins alter adipose tissue function and increase inflammation; therefore, HIV-associated chronic inflammation and immune activation may play a direct role in the development of lipohypertrophy [5].

The risk of developing lipodystrophy is greater with advanced age, in people who have the longest durations of infection and in patients who have had therapy for longer periods [3,4]. Furthermore, some studies have shown that patients with lipoatrophy have significantly lower BMI than those with lipohypertrophy [2,8].

The combination of obesity, lipodystrophy, central adiposity, dyslipidemia, and insulin resistance commonly occurs among HIV-infected adults; these constitute risk factors associated with cardiovascular diseases, which represent the third most common cause of death among HIV-positive individuals in Europe. Moreover, lipodystrophic body changes can influence the quality of life, leading to low adherence to HAART and subsequent virologic and clinical failure [3,9,4,5].

In this case, lipodystrophy syndrome was a complicated obstacle to the necessary intervention. Surgery, despite all of its technical difficulties, was the only method to obtain the diagnosis considering the failed aspiration attempts. Moreover, the patient had shown progression of thyrotoxicosis, which justified the choice of surgical intervention. The surgical indication was correct because the histological examination was positive for papillary carcinoma.

In conclusion, we believe that every medical or surgical intervention must be aimed at improving or maintaining the patient’s health and quality of life; therefore, we must try to overcome technical complexity where possible. The collaboration of an experienced team is essential to obtain satisfactory results, especially in the most challenging cases.

## Declaration of Competing Interest

The authors have no conflicts of interest to disclose.

## Funding

No funding

## Ethics approval

For single case report NO ethical approval needs. Patient signed a consent for publishing the case report.

## Consent

Patient signed a consent for the publication of this case report.

## Author contribution

EC and BA wrote the case report. AS and UM revised the case report.

## Registration of Research Studies

Ethical Board approval is not required for case reports in our Center.

## Guarantor

Prof. Uliano Morandi is the Guarantor of this case report.

## Provenance and peer review

Not commissioned, externally peer-reviewed
